# Differences in Patient Experiences Among People From Ethnic Minority Backgrounds: A Comparative Analysis of HCAHPS Results

**DOI:** 10.1177/23743735231218867

**Published:** 2023-12-07

**Authors:** Corey Adams, Anthony Schembri, Ashfaq Chauhan, Ramesh Walpola, Reema Harrison

**Affiliations:** 1 208044 Centre for Health Systems and Safety Research, Australian Institute of Health Innovation (AIHI), Macquarie University, Sydney, Australia; 2 2784 St Vincent's Health Network Sydney, Sydney, Australia; 3 School of Health Sciences, 7800 University of New South Wales (UNSW), Australia

**Keywords:** patient experience, measurement, inclusion, ethnic minority, HCAHPS

## Abstract

Patients from ethnic minority backgrounds often experience disparities in healthcare quality and outcomes. This study aimed to compare the patient-reported experiences of patients with limited English proficiency (LEP) to general patients in the Australian healthcare setting. The Hospital Consumer Assessment of Healthcare Providers and Systems (HCAHPS) survey was used to evaluate patient experiences from patients in a metropolitan public healthcare network, spanning three hospitals. Level of English proficiency was based on primary language spoken at home. To identify disparities in experience ratings between patients with LEP and the general cohort, independent *t*-tests were employed. Data was analysed from 2,291 patients, collected over a five-year period (2017–2022), with 490 patients identified as LEP (i.e. speaking a language other than English at home). Statistically significant differences were identified between the cohorts, with LEP patients rating their experiences higher in three areas: doctors listening carefully, doctors explaining in a way they could understand, and quietness at night. Conversely, patients with LEP scored lower in areas regarding nursing respect and responsiveness to call bells. Although patients with LEP had a more positive overall experience, this difference was not statistically significant. The findings indicate potential misalignment between the often poorer health outcomes among people from ethnic minority backgrounds and their experiences in hospital. Additional research is crucial to delve into the unique experiences of ethnic minority patients, including those with LEP, to understand the differences influencing perceptions of care and contributing to disparities in health outcomes.

## Key Findings

- The focus on general patient experience ratings may obscure valuable insights about the experiences of specific patient cohorts, including those from ethnic minority backgrounds and LEP.
- The results from this study indicated that patients with LEP expressed positive healthcare experiences across multiple domains, particularly in communication with doctors; however scored lower in other HCAHPS domains, such as respect from nurses and staff responsiveness to call bells.
- These findings suggest misalignment between experience ratings and quality indicators for patients from ethnic minority backgrounds.
- To better understand the experiences of patients with LEP, culturally relevant questions may need to be developed and incorporated into patient experience measurement tools.

## Introduction

Enhancing patient experience of care is crucial for establishing high-performing health systems and is acknowledged as a core component of the Quadruple Aim of healthcare.^1^ Information about patient experiences may provide valuable insights to guide improvements that elevate the quality, outcomes and equity of healthcare services.^2^ Accordingly, health organisations routinely collect data on patient experiences to drive quality improvement projects, which is further bolstered by policy directives that promote active consumer involvement in both the design and assessment of health services.^3^ In the context of patient experiences and safety, individuals from ethnic minority backgrounds frequently experience poorer care outcomes when compared to the general population, including increased risk for patient safety events.^3–5^ Addressing these disparities is crucial for improving healthcare equity. One method includes leveraging patient-reported experience and outcomes data to guide targeted improvement activities.^6^

Tools for measuring patient experience, like the Hospital Consumer Assessment of Healthcare Providers and Systems (HCAHPS) surveys, are commonly employed for assessing patient experience. However, only a limited number of these tools extensively explore experiences related to ethnicity. Research into patient experience data has revealed disparities in primary and tertiary care among different ethnic groups. For instance, in the United States, analysis of 1.2 million HCAHPS surveys revealed that patients of Asian and Hispanic descent rated their experiences lower compared to those of non-Hispanic white patients.^7^ Similarly, a UK-based analysis of the 2009 English General Practice Patient Survey, consisting of 11 patient experience measures, reported fewer positive experiences for South Asian and Chinese patients compared to White patients.^8^ Differences between African American and White respondents, however, were minimal.^8^ Studies indicate that drivers of patient experience vary significantly according to patient ethnicity, primarily in aspects such as differences in communication with doctors and nurses, and in the courtesy and respect demonstrated by staff.^7,9,10^ Therefore, more research is required to identify and mitigate the barriers related to inequities in healthcare.

Patients who have limited English proficiency (LEP) often face lower quality of care and increased risks, including higher incidents of physical harm, due to communication errors.^5,11^ Studies have found that patients with LEP often do not receive healthcare information as effectively as those who are fluent in English.^12^ This may be influenced by the frequent use of family members as informal interpreters, healthcare staff dedicating less time to patients with LEP, and the tendency to convey substantial information rapidly when professional interpreters are used, potentially overwhelming the patients’ understanding.^12^

In Australia, the nation's diversity is prominently reflected in the demographics of its population. The 2016 Census, as reported by the Australian Bureau of Statistics, reveals that over one-fifth (21%) of the population speaks a language other than English within their homes,^13^ thereby highlighting the linguistic diversity within Australia. The Australian Government endorses the employment of professional interpreters, especially for patients with LEP. This policy is crucial for ensuring that these individuals receive clear and accurate information during critical communications and decision-making processes, however research indicates some challenges in the practical implementation of these policies in healthcare settings.^12^

This study intends to examine and compare the experiences of patients with LEP against those of the broader patient population within public healthcare systems, aiming to understand the existing disparities among patients from diverse ethnic backgrounds. Furthermore, it will investigate how effectively the HCAHPS survey captures the experiences of patients with LEP.

## Method

### Ethics

Ethical approval was provided by the participating organisation's local Human Research Ethics Committee.

### Study Design

A cross-sectional observational study was conducted.

### Participants and Setting

The study examined the experiences of patients admitted to a major public healthcare network in Sydney, Australia, which encompasses three metropolitan hospitals. Collectively, these hospitals accommodate more than 500 beds and receive upwards of 8,000 patient admissions each year.^14^

### Materials

Patient experience was evaluated using the HCAHPS survey, which was routinely used in the healthcare setting to measure patient experience (evaluated biannually). The HCAHPS survey has been mandated for patient experience measurement in the United States and has been rigorously tested and validated for use.^15,16^ The HCAHPS survey consists of 22 questions spanning nine different domains: communication with nurses, communication with doctors, response of hospital staff, hospital environment, communication about pain, communication about medicines, pain management, discharge information and care transitions. Responses can be rated on a five-point Likert scale, which ranges from ‘Never’ to ‘Always’. To facilitate completion from patients of diverse ethnic backgrounds, the HCAHPS surveys were available in various languages – including English, Spanish, Chinese, Russian, Vietnamese, Portuguese, German, Tagalog and Arabic.

### Procedure

Patients were sent a copy of the HCAHPS survey within four weeks of discharge from the hospital, either via email or in paper-based format. Participation in the study was voluntary, and consent was implied with completion of the survey.

### Data Analysis

Patients were classified as having LEP if their primary language was identified as ‘other than English’. Secondary analysis of routinely collected HCAHPS data was conducted using independent samples *t*-tests in IBM SPSS Statistics (Version 25). This method enabled the identification of statistically significant differences in various aspects of patient experiences between general patients and those with LEP.

## Results

During the five-year study period (2017–2022), 2,291 patients responded to the survey, reflecting a 21% response rate. Among the respondents, 490 people (or 22%) indicated a primary language other than English, and thereby were categorised as patients with LEP. As shown in Table 1, the demographic breakdown of respondents was 57.4% male (1,316/2,291), with a mean age of 65.3 years.

**Table 1.** HCAHPS Respondent Characteristics.

Characteristic (primary language)	English	Other than English	Total
**Language spoken at home**	1,801 (88%)	490 (22%)	2,291 (100%)
**Sex**			
Male	1,040	276	1,316 (57.4%)
Female	761	214	975 (42.6%)
**Age group in years**			
<20	12	4	16 (0.7%)
20–39	151	47	198 (8.6%)
40–59	436	100	536 (23.4%)
60–79	841	208	1,049 (45.8%)
80+	361	131	492 (21.5%)

The ratings to HCAHPS questions for patients with LEP and the general population are outlined in Table 2. An independent samples *t*-test was conducted to compare the patient-reported experience measures between the general population and patients with LEP. Levene's test was used to test the assumption of homogeneity of variance, which indicated *p* < .001, hence equal variance was not assumed.

**Table 2.** Respondent Ratings to HCAHPS Questions.

Questions	Primary language	*N*	Mean	Std. deviation
*What number would you use to rate this hospital during your last stay? (0–10)*	English	1,699	9.69	1.769
	Other	490	9.84	1.771
*Would you recommend this hospital to your friends and family?*	English	1,616	3.71	0.606
	Other	471	3.73	0.578
*How often did nurses treat you with courtesy & respect?* ^a^	English	1,693	3.83	0.444
	Other	489	3.75	0.529
*How often did nurses listen carefully to you?*	English	1,685	3.63	0.618
	Other	481	3.64	0.636
*How often did nurses explain in a way you could understand?*	English	1,676	3.60	0.654
	Other	481	3.59	0.690
*After you pressed the call bell, how often did you get help as soon as you wanted it?* ^a^	English	1,413	3.26	0.766
	Other	480	3.08	1.052
*How often did doctors treat you with courtesy & respect?*	English	1,687	3.82	0.468
	Other	485	3.84	0.453
*How often did doctors listen carefully to you?* ^b^	English	1,674	3.66	0.662
	Other	480	3.76	0.532
*How often did doctors explain things in a way you could understand?* ^a^	English	1,674	3.62	0.663
	Other	479	3.72	0.557
*How often were your room and bathroom kept clean?*	English	1,687	3.61	0.676
	Other	484	3.62	0.610
*How often was the area around your room quiet at night?* ^b^	English	1,669	2.93	0.889
	Other	479	3.14	0.907
*How often did you get help using the bathroom or in using a bedpan as soon as you wanted?*	English	731	3.32	0.804
	Other	242	3.36	0.849
*Before giving you new medicine, how often did hospital staff tell you what the medicine was for?*	English	1,145	3.49	0.829
	Other	328	3.48	0.805
*How often did staff describe possible side effects in a way you could understand?*	English	1,129	2.93	1.153
	Other	323	2.95	1.144
*Staff took my preferences and those of my family or caregiver into account in deciding what my health care needs would be when I left.*	English	1,618	3.26	0.760
	Other	472	3.29	0.749
*When I left the hospital, I had a good understanding of the things I was responsible for in managing my health.*	English	1,649	3.37	0.709
	Other	477	3.38	0.727
*When I left the hospital, I clearly understood the purpose for taking each of my medications.*	English	1,559	3.57	0.698
	Other	464	3.58	0.659

a Significantly more negative scores for patients with LEP.

b Significantly more positive scores for patients with LEP.

### Differences in HCAPS Ratings People From Ethnic Minority Backgrounds

Patients with LEP reported a higher overall experience rating (*M* = 9.84, SD = 1.77) than general patients (*M* = 9.69, SD =1.76). However, this difference was not statistically significant (p = 0.113).

When comparing the HCAHPS ratings of patients with LEP to the general population, three questions had significantly higher scores and two questions were significantly lower scoring for patients with LEP. In the domain of communication with doctors, patients with LEP scored significantly higher for the question ‘*How often did doctors listen carefully to you?*’ (*M* = 3.76, SD = 0.53) compared to the general population (*M* = 3.66, SD = 0.66), *t*(946) = -3.46, *p* = .001. Similarly, the question ‘*How often did doctors explain things in a way you could understand?*’ had higher scores for patients with LEP (*M* = 3.73, SD = 0.55) than general patients (*M* = 3.62, SD = 0.66), *t*(901) = -2.997, *p* = .001. Additionally, there was a significantly higher rating for the question regarding ‘*How often was the area around your room quiet at night?*’ for patients with LEP (*M* = 3.14, SD = 0.907) than general patients (*M* = 2.93, SD = 0.88), *t*(761) = -4.57, *p* = .000. With regards to lower scoring domains, there was a significantly lower ratings provided by patients with LEP for two nursing-related questions: ‘*How often did nurses treat you with courtesy and respect?*’ (*M* = 3.75, SD = 0.53) compared to general patients (*M* = 3.83, SD = 0.44, *t*(698.07) = 2.73, *p* = .006, and ‘*After you pressed the call bell, how often did you get help as soon as you wanted it?*’ for patients with LEP (*M* = 3.08, SD = 1.05) compared to general patient ratings (*M* = 3.26, SD = 0.76); *t*(659) = 3.42, *p* = .001.

## Discussion

The findings from this study reveal that patients with LEP generally reported positive hospital experiences via the HCAHPS surveys. However, these findings also illustrate that there may be potential limitations when using HCAHPS to evaluate patient experience in patients from culturally and linguistically diverse backgrounds. In particular, patients with LEP rated significantly higher for three questions in the HCAHPS survey, and significantly lower for two questions, compared to general patients. Whilst these findings demonstrate positive experiences for patients with LEP, they contrast with the disparities in safety and quality health outcomes noted for culturally diverse patients.^3^ As such, these results suggest potential misalignment between experience ratings and quality indicators for patients from culturally and linguistically diverse backgrounds.

Of the three HCAHPS questions that received higher scores from patients with LEP, two questions related to communication with doctors (‘How often did doctors listen carefully to you?’ and ‘How often did doctors explain things in a way you could understand?’). Conversely, the two HCAHPS questions that received lower scores for LEP patients primarily related to interactions with nurses, specifically concerning nursing respect and staff responsiveness to call bells. These findings support previous research about HCAHPS differences for ethnic minority groups, which identified the most pronounced variation in the domains of doctor and nurse communication, as well as pain management.^7,10^ Additionally, the findings of this study also highlighted the importance of nurse empathy and respect. There may be important differences relating to specific cultures that impact HCAHPS ratings. For example, studies have reported that Asian American patients placed less importance on the effectiveness of communication, and more emphasis on staff courtesy and respect than general patients.^9^

These findings may reflect variances in the expectations of care quality by patients from ethnic minority backgrounds and the gratitude they expressed for the care they received. In general, cultural differences may bring about specific social norms and expectations; therefore individuals may perceive heightened satisfaction with the healthcare they receive, even if the quality of care is not superior to that provided to the general population.^17^ This phenomenon, known as ‘the Happy Migrant Effect’, describes situations whereby patients express satisfaction with healthcare services, despite issues in service provision. Various factors may influence this sentiment, including cultural tendencies toward social desirability, favourable comparisons to healthcare in their native countries, and/or apprehensions about potential consequences from voicing complaints.^17^

Additionally, these findings highlight the importance of language proficiency. Existing research indicates that patients with LEP may encounter communication barriers in healthcare, yet may not be view these situations negatively.^18^ For example, a study on Spanish-speaking LEP patients identified irregularities in call bell responsiveness from healthcare staff. Despite this, patients with LEP initially denied communication issues, feeling competent in basic conversations, and were comforted by the presence of friends and family.^19^ Similarly, an Australian study revealed that immigrant patients often expressed positivity towards healthcare experiences, in spite of cultural barriers, due to immense gratitude for the care they received.^20^ Therefore, the actual influence of language barriers may not be completely reflected in typical patient experience surveys, including HCAHPS. These findings highlight the importance of conducting more thorough research in this area and exploring different evaluation techniques to measure the experience of patients from ethnic minority backgrounds.

Whilst our findings indicate predominantly positive healthcare experiences for patients with LEP, these results may reflect the specific sample or hospitals under study. Another possibility is that the structure and subjective interpretation of HCAHPS questions do not adequately address the culturally and linguistically relevant topics for patients. Moreover, variations in patient experiences may be rooted in culturally influenced perceptions of healthcare roles. For instance, some patients may inherently regard doctors with higher reverence compared to other healthcare practitioners, potentially skewing their patient experience ratings.^21,22^ Notably, in our study, only one nursing domain question scored lower for patients with LEP, with no significant differences regarding communication with nurses between ethnic minorities and general patients. As such, further information is required to adequately understand the context and quality of these interactions between patients and clinicians, especially nurses.

Whilst tools like the HCAHPS can be useful for assessing patient experiences, they also present limitations when it comes to people from ethnic minority backgrounds. Accordingly, quantitative surveys may not capture all of the issues and topics that are most important to patients with LEP. For instance, deeper insights may be required about responsiveness to call bells to determine whether the ratings are indicative of actual delays in clinician response, or a reflection of varying expectations associated with cultural differences. Therefore, to sufficiently understand the experiences of patients from ethnic minority backgrounds, healthcare organisations may need to utilise a more nuanced and culturally sensitive approach, which extends beyond translating survey questions into multiple languages.

### Limitations

There are several limitations to the present study that should be acknowledged. Firstly, the data was collected from only one healthcare network, which might limit the generalisability of the findings. As such, future research should involve a larger sample size and multiple hospitals to provide a more comprehensive understanding of the experiences of ethnic minority patients across different healthcare settings. Also, this study highlighted the lack of demographic information collected during routine patient experience measurement (such as country of birth and proficiency of English), which may have impacted the ability to identify participants from ethnic minorities. The collection of additional demographics, such as comorbidities and length of stay, may support statistical analysis using linear regression. Therefore, future research should focus on gathering more comprehensive demographic data, facilitating the more precise identification of patients from ethnic minorities and their medical histories. Additionally, future research may also examine differences between specific cultural groups; for example, examining differences in patient-reported measures pertaining to country of birth.

### Implications for Research, Policy and Practice

Recognising and addressing the distinct experiences of ethnic minority patients can lead to more equitable healthcare systems and improved health outcomes. Although the generally positive outcomes of this study might be attributed to the selected sample and healthcare network, it is also possible that these findings are influenced by the way in which experience questions are framed, potentially overlooking culturally relevant issues. Consequently, further research might be needed to assess whether existing measures effectively capture the experiences of patients with LEP. For instance, this could be achieved by reviewing the results across a range of healthcare measures and using analysis, such as Delphi technique, to determine the relevant items and framing. Additionally, healthcare providers may consider developing and implementing targeted patient experience measurement tools, with additional questions that address the unique needs and perspectives of people from ethnic minority backgrounds.

Furthermore, the practical implications illustrate the need to ensure that all patients can provide feedback about their healthcare experiences. By considering the inherent cultural complexities of patient experience measurement, healthcare organisations may benefit from complementing survey data with other methods of evaluation, such as interviews and focus groups. A multi-faceted approach to patient experience measurement may provide a more detailed and more accurate understanding about the diversity of patient experiences. Finally, given the emphasis these findings place on clinician communication, enhancing healthcare staff training in effective communication skills and cultural sensitivity could help them more successfully meet the needs of patients from ethnically diverse backgrounds and those with LEP.

## Conclusion

This study examined disparities in patient experiences between those with limited English proficiency (LEP) and the general patient population within the Australian healthcare context, using HCAHPS patient-reported measures for analysis. Although patients with LEP reported positive experiences in various aspects, especially in communication with doctors, their feedback also highlighted areas needing improvement, particularly with nursing respect and staff responsiveness. The results of our study suggest misalignment between experience ratings and quality indicators for patients from ethnic minority backgrounds. However, healthcare providers can adopt more flexible evaluation methods, such as developing tailored tools specifically for culturally diverse patients. This approach would enable a more profound and comprehensive understanding about the experiences of patients with LEP. These insights are critical in directing the development of healthcare improvements, ultimately leading to better care quality and enriched experiences for patients from culturally and linguistically diverse backgrounds.
